# Trends in carbapenem resistance in Pre-COVID and COVID times in a tertiary care hospital in North India

**DOI:** 10.1186/s12941-022-00549-9

**Published:** 2023-01-03

**Authors:** Nirupama Chatterjee, Pushpa K. Nirwan, Shruti Srivastava, Ruchi Rati, Lalit Sharma, Priyanka Sharma, Priyambada Dwivedi, Namita Jaggi

**Affiliations:** 1 Education and Research, Artemis Hospitals, Sector-51, Gurugram, Haryana India; 2 Department of Microbiology, Artemis Hospitals, Sector-51, Gurugram, Haryana India

**Keywords:** Carbapenem resistance, COVID, NDM, OXA-48 family, India

## Abstract

**Background:**

Carbapenem resistance is endemic in the Indian sub-continent. In this study, carbapenem resistance rates and the prevalence of different carbapenemases were determined in *Escherichia coli, Klebsiella pneumoniae, Acinetobacter baumannii, Pseudomonas aeruginosa* during two periods; Pre-COVID (August to October 2019) and COVID (January to February 2021) in a north-Indian tertiary care hospital.

**Methods:**

Details of patient demographics and clinical condition was collated from the Hospital Information System and detection of carbapenemases NDM, OXA-48, VIM, IMP and KPC was done by Polymerase chain reaction (PCR) in 152 and 138 non-consecutive carbapenem resistant isolates during the two study periods respectively. Conjugation assay and sequencing of NDM and OXA-48 gene was done on a few selected isolates.

**Results:**

As compared to Pre-COVID period, co-morbidities and the mortality rates were higher in patients harbouring carbapenem resistant organisms during the COVID period. The overall carbapenem resistance rate for all the four organisms increased from 23 to 41% between the two periods of study; with *Pseudomonas aeruginosa* and *Klebsiella pneumoniae* showing significant increase (p < 0.05). OXA-48, NDM and co-expression of NDM and OXA-48 were the most common genotypes detected. NDM-5 and OXA-232 were most common variants of NDM and OXA-48 family respectively during both the study periods.

**Conclusion:**

Higher rate of carbapenem resistance in COVID times could be attributed to increase in number of patients with co-morbidities. However, genetic elements of carbapenem resistance largely remained the same in the two time periods.

**Supplementary Information:**

The online version contains supplementary material available at 10.1186/s12941-022-00549-9.

## Background

Carbapenems are one of the last resort antibiotics for the treatment of infections caused by multidrug-resistant Gram-negative bacteria. With the increasing use of carbapenems in the clinical settings, the emergence and spread of resistance to these antibiotics constitutes a major public health concern, especially in Indian subcontinent where the rates of antibiotic resistance in general, remain high due to burden of infectious diseases, over-use of antibiotics and poor sanitation conditions [1, 2].

The pandemic COVID-19 was anticipated to have multilayered and long-term effects on antibiotic resistance pattern. Measures like social distancing, increased compliance to hand hygiene protocols and drop in international travels were presumed to limit the transmission of resistance genes. Countering these beneficial aspects, the overuse of antibiotics for fear of secondary bacterial infections and decreased seriousness about antimicrobial stewardship could have an adverse effect on the resistance rates [3, 4].

The present study is an attempt to understand the effect of COVID-19 pandemic on carbapenem resistance rates in a north Indian tertiary care hospital. The hospital caters to both Indian and international patients; but during COVID times there was a steep reduction in the latter group. Besides, the patient flow in the hospital during COVID pandemic followed a pattern different from Pre-COVID times, with lowered footfall in the initial lockdown period and slow return to normal after the lockdown. These factors are expected to have an effect on the carbapenem resistance rates which are spread mainly by plasmid based carbapenemases genes. Of the five carbapenemases genes NDM, OXA-48, VIM, IMP and KPC that are widely detected world-wide, the first two are most commonly reported from northern India at present [5, 6].

We checked the carbapenem resistance rates in four clinically important Gram negative species; *Escherichia coli*, *Klebsiella pneumoniae*, *Acinetobacter baumannii*, *Pseudomonas aeruginosa* during two time periods Pre-COVID (August to October, 2019) and during COVID period (January to February 2021). Further, we assessed the prevalence of different carbapenemases in the carbapenem resistant isolates. The variant of NDM and OXA-48 family present in the isolates and the plasmid backbone were determined in few selected isolates. Knowledge of the prevalent carbapenemases and their genetic background helps in judicious choice of antibiotics and unfolding the transmission route of genes. The results of this study will help in deciding empiric antibiotic therapy, designing new protocols for infection control and surveillance in the hospital and thus bringing down the rate of carbapenem resistance.

## Methods

### Bacterial culture, species identification and antibiotic susceptibility

The study was carried out at a tertiary care referral hospital in Gurugram, North India in two phases: Pre-COVID (August to October 2019) and COVID (January to February 2021). The study has been passed by the Institutional Ethics Committee (ECR/53/Inst/HR/2013/27Apr2016). In our routine practice; clinical samples, such as urine, sputum, blood, pus, tracheal aspirate, and body fluids received in the clinical microbiology department from both inpatient [including intensive care unit (ICU)] and outpatient departments are cultured by following standard bacteriological techniques used for the isolation of the organisms [7]. Identification of isolates and antibiotic susceptibility testing was performed on automated Vitek-2 compact system (Biomerieux, France). Carbapenem resistance was decided on the basis of minimum inhibitory concentration (MIC) values as determined by VITEK system and interpreted according to CLSI guidelines [8].

### Patient demographics and other details

Details of patients’ sex, age, nationality, location, co-morbidities, mortality and length of stay were collated from the Hospital Information System.

### Molecular detection of carbapenemases

One hundred fifty-two and one hundred thirty-eight non-duplicate, non-consecutive carbapenem resistant isolates of four Gram-negative species; *Escherichia coli*, *Klebsiella pneumoniae*, *Pseudomonas aeruginosa* and *Acinetobacter baumannii* from the two study periods (Pre-COVID and COVID period) respectively were screened for the detection of carbapenemases NDM, OXA-48 family, VIM, IMP and KPC by PCR. DNA was extracted by boiling lysis method and the PCR conditions consisted of initial denaturation at 95 °C for 5 min followed by 35 cycles of denaturation, annealing and extension for one minute each at 95°C, 55°C and 72°C respectively. The primers used for NDM, VIM, IMP and KPC are as described before [9, 10], while the sequence of OXA-48 family primer is as follows (Forward Primer: GATTATC GGAATGCCAGCGG Reverse primer: CCCTAAACCATCCGATGTGG). The OXA-48 primers were designed using Primer 3 software (NCBI primer blast) and covered the conserved regions of OXA-48 family. PCR products of 19 NDM positive and 9 OXA-48 positive isolates of each period were randomly selected and sequenced to determine the particular variant of NDM and OXA-48. The sequences were compared with known NDM and OXA-48 family variant sequences from Genbank, NCBI using the NCBI BLAST software (https://blast.ncbi.nlm.nih.gov).

### NDM gene location and transmissibility

Location of NDM gene (chromosomal/plasmid) was determined using conjugation assay. Conjugation was done by liquid mating as described before with some modifications [11, 12]. In liquid mating, overnight cultures of donor and recipient *E. coli J53* (Azide^R^) were mixed in the ratio of 1:2 and incubated without shaking at 37 °C for 6 h. After stopping the process of conjugation by pulse vortexing, the culture was spread on plate containing sodium azide (200mg/L) and meropenem (8µg/ml). The presence of NDM and OXA-48 in trans-conjugants was confirmed by PCR.

### Plasmid profiling by restriction digestion

Eight plasmids containing NDM or NDM + OXA-48 from the COVID period were subjected to restriction digestion by EcoR1. Plasmid was extracted using the commercial kit (QIAprep Spin Miniprep Kit, Qiagen cat#27104) followed by quantification using spectrophotometer (Biophotomter, Eppendorf). Around 1000ng of plasmid was digested using EcoR1 (Thermo, Cat#ER0271) for 3h at 37°C following manufacturer’s protocol. The digested plasmid was run on 1% agarose at 60 V for 7 h.

### Statistical analyses

Continuous variables have been presented in the form of Mean ± S.D./ Median(I.Q.R.) and categorical variable have been presented in the form of frequency and percentages. Qualitative variables were correlated using Chi-Square test /Fisher’s exact test. Means for independent variable were compared using the Independent t-test/Mann–Whitney test. Adjusted odds ratio has been calculated with the help of Binary Logistic Regression. Odds ratio has been adjusted for all baseline characteristics such as age, sex, nationality, location, length of hospital stay. p values were calculated 2 sided and p < 0.05 was considered statistically significant. All analyses have been done using the software SPSS Ver.21.

## Results

### Distribution of organisms and carbapenem resistance rates

**Fig. 1:**
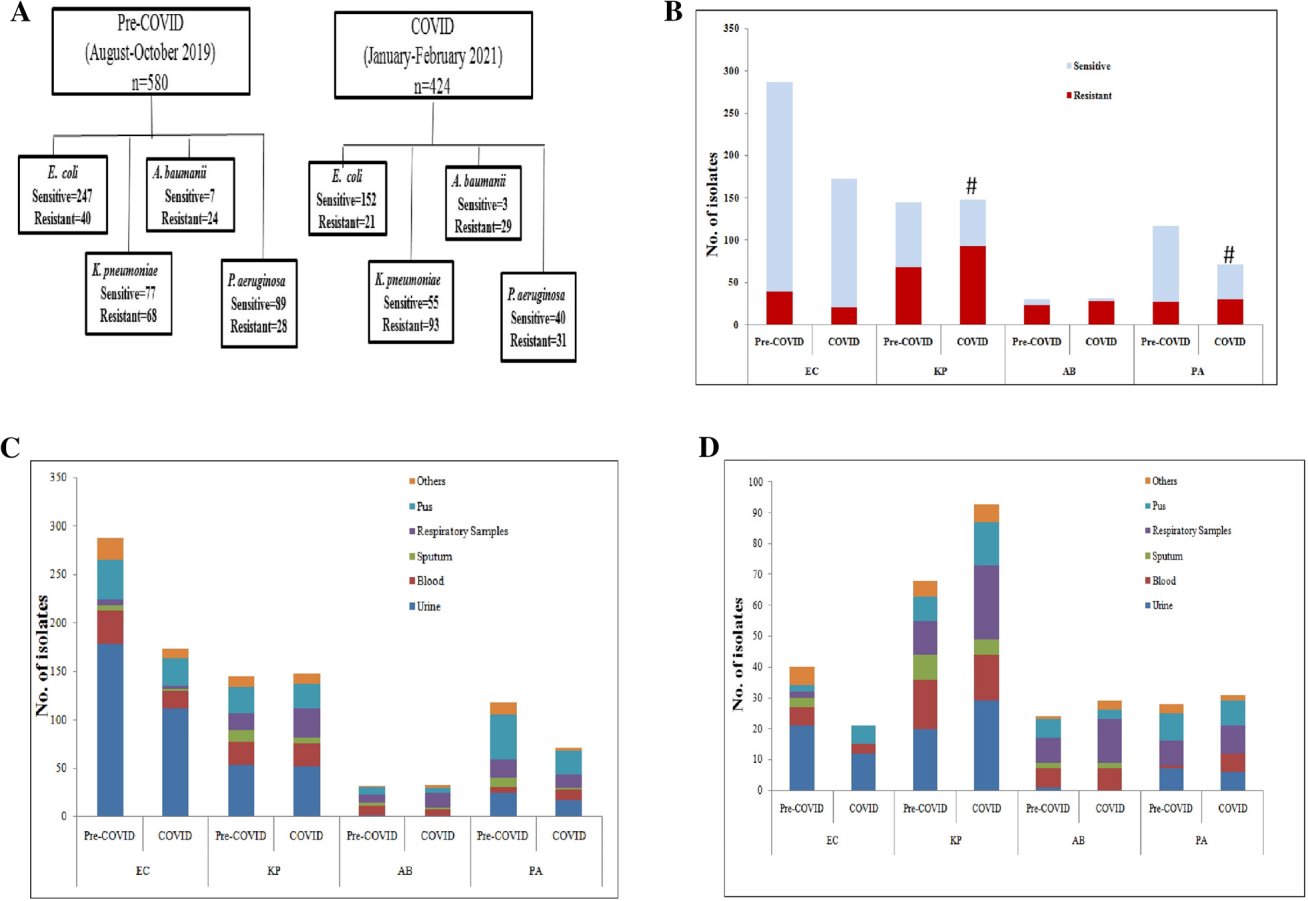
Carbapenem resistance rates in Pre-COVID and COVID times. **A** Flow chart showing the distribution of organisms of the four species: *E. coli*, *K. pneumoniae*, *A. baumannii* and *P. aeruginosa* during Pre-COVID and COVID times and their resistance rates. **B** Carbapenem resistance rates in the four gram negative species under study: *E. coli*, *K. pneumoniae*, *A. baumannii* and *P. aeruginosa* during Pre-COVID and COVID times. #indicates significant difference (p < 0.05) in resistance rates between the two phases of study (independent t-test). **C** Distribution of all the isolates (Sensitive and Resistant) of *E. coli*, *K. pneumoniae*, *A. baumannii* and *P. aeruginosa* in different sample types during Pre-COVID and COVID times. *Note: Respiratory samples include bronchoalveolar lavage and Tracheal aspirate*. D: Distribution of only resistant isolates of *E. coli*, *K. pneumoniae*, *A. baumannii* and *P. aeruginosa* in different sample types during Pre-COVID and COVID times. EC: *E. coli*, KP: *K. pneumoniae*, AB: *A. baumannii*, PA: *P. aeruginosa*, Pre-COVID: August to October 2020; COVID: January to February 2021

The total number of isolates received in the clinical microbiology lab for the species under study: *E. coli*, *K. pneumoniae*, *P. aeruginosa* and *A. baumannii* during the first phase (August to October 2019) and second phase (January to February 2021) were 580 and 424 respectively. Of these 160 (27%) and 174 (41%) were carbapenem resistant during the first and second phase respectively. The maximum numbers of resistant isolates were of *K. pneumoniae* forming 48.5% and 53.4% of the total resistant samples in the Pre-COVID and COVID periods respectively. However, in terms of carbapenem resistance rates, the maximum resistance rate was observed in *A. baumannii* followed by *K. pneumoniae*, *P. aeruginosa* and *E. coli* in that order during both the time periods. Overall, urine was the most common sample and also the dominant sample type in *K. pneumoniae* and *E. coli*. Pus was the second most common sample type and was the dominant sample type in *P. aeruginosa*. Bacteremia was caused by all the four species, with *E. coli* and *K. pneumoniae* being the most dominant species during the first and second phase respectively. The percentage of respiratory samples (bronchoalveolar lavage and endotracheal aspirate) increased from 8.9% in pre-COVID times to 14.8% during COVID times. The resistance rates for all the four organisms and their distribution in different sample types has been shown in Fig. 1A–D. The carbapenem resistance rates of *P. aeruginosa* and *K. pneumoniae* showed a significant increase from Pre-COVID to COVID times (p < 0.05).

Almost 50% of the *A. baumannii* isolates were received from the ICUs in both the phases, where as for *K. pneumoniae* these percentages were 33% and 37% in the first and second phases respectively. In case of *E. coli* and *P. aeruginosa*, the percentages of isolates received from ICU were less than 20% during both the study periods.

*Demographic and other details of patients harboring carbapenem resistant organism:* Age did not have any significant effect on carbapenem resistance rates. The difference in the carbapenem resistance rates of elderly patients (age ≥ 60) and other patients (age < 60) was not statistically significant (p > 0.05). Isolates which were found to be carbapenem resistant were almost twice as likely to be from male patients during the pre-COVID times, but this difference was not significant during COVID times. Location wise, maximum number of carbapenem resistant isolates was reported from the Intensive Care Units (ICUs) during both the time periods. Co-morbidities including neoplasia, immune-compromised conditions like diabetes, rheumatism, infections like tuberculosis and hypertension etc. made individuals more susceptible to carbapenem resistance during COVID times(p < 0.05), but the same was not true for the Pre-COVID times. We also observed a significant rise in percentage of patients with co-morbidities during the COVID times (p < 0.05). Amongst the co-morbidities, patients with neoplasia comprised about 12%; neurological conditions and chronic kidney disease at around 5% of the total patients during both the time periods. However, there was slight increase from 9 to 11% in patients suffering with autoimmune disease like diabetes, hypertension and chronic infections like tuberculosis and hepatitis B from the Pre-COVID to COVID period. The percentage of patients with coronary artery disease increased from around 2% to 6% from Pre-COVID to COVID times. An interesting observation was the difference in resistance rates in the various co-morbidities group during the two phases of the study. Whereas, during the pre-COVID phase there was no significant difference in the carbapenem resistance rates in any of the groups of co-morbidities; during the COVID times resistance rate was significantly high in patients with neoplasia and chronic kidney disease. In the second phase, 15 patients of the total 331 in our sample were suffering from COVID and related complications of which 12 (80%) harbored carbapenem resistant bacteria. The data suggests that COVID and related complications lead to increased susceptibility to carbapenem resistance (p < 0.05). The length of stay was significantly higher in patients harboring carbapenem resistant as compared to sensitive organisms during the COVID time period (p < 0.05). Patients harboring carbapenem resistant organisms had higher mortality rates than sensitive group during both the study phases (p < 0.05). Details of demographics and other details are shown in Table 1.

**Table 1:** Demographics and clinical details of patients

	2019				2021			
	Sensitive (336)	Resistant (112)	Adjusted odds ratio [CI]	p-value	Sensitive (201)	Resistant (116)	Adjusted odds ratio[CI]	p-value
Patients Age ≥ 60(%)	146 (43.5)	67 (59.8)	1.64 [0.95, 2.82]	0.07	112 (55.7)	60 (52.2)	0.67 [0.39, 1.17]	0.16
Sex Male (%)	170 (50.5)	75 (66.9)	1.82 [1.05, 3.14]	0.03	110 (54.7)	71 (61.2)	0.99 [0.57, 1.72]	0.98
Nationality International patients (%)	52 (15.4)	18 (16)	2.20 [1.08, 4.48]	0.03	12 (5.9)	9 (7.8)	1.71 [0.59, 4.97]	0.32
Location IPD vs. OPD	108 (32.1)	25 (23)	2.86 [1.31, 6.23]	0.01	65 (32.3)	22 (19.1)	0.75 [0.35, 1.60]	0.36
ICU vs. OPD	31 (9.2)	69 (61)	24.38 [10.62, 55.98]	< 0.001	32 (15.9)	66 (57.3)	3.46 [1.64, 7.29]	< 0.001
Co-morbidities (%)	117 (34.8)	54 (48.2)	1.11 [0.64, 1.92]	0.72	96 (47.7)	76 (65.5)	2.88 [1.65, 5.02]	0.00002
Length of stay Days 2–7 vs. Day 0–1	102 (30.4)	34 (30.4)	0.676 [0.304, 1.501]	0.336	79 (39.3)	37 (32.2)	1.51 [0.7, 3.22]	0.29
Days > 7 vs. Day 0–1	60 (17.9)	55 (49.1)	1.26 [0.563, 2.82]	0.574	39 (19.4)	59 (51.3)	2.62 [1.13, 6.08]	0.02
% Mortality	5.4	18	2.36 [1.11, 5.01]	0.03	5	26.1	3.08 [1.31, 7.26]	0.01

### Prevalence of different carbapenemases and variants of NDM, OXA-48 family

**Fig. 2:**
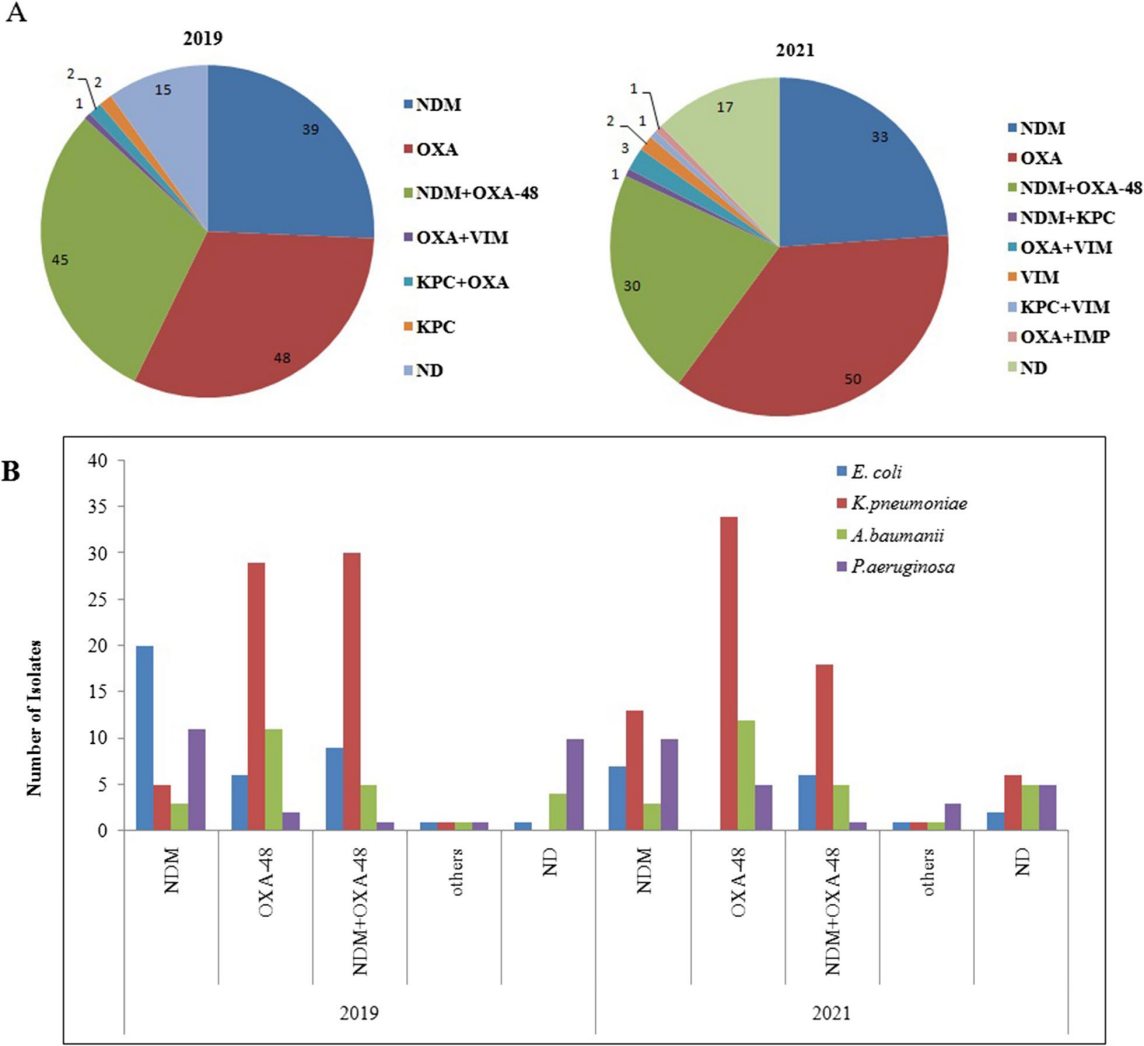
Frequency and distribution of various carbapenemases in the isolates. **A** Frequency of the common carbapenemases in the total number of resistant isolates during the two phases of the study. **B** Organism wise distribution of the common carbapenemases during the two phases of the study. EC: *E. coli*, KP: *K. pneumoniae*, AB: *A. baumannii*, PA: *P. aeruginosa*, Pre-COVID: August to October 2020; COVID: January to February 2021

OXA-48, OXA-48 in combination with NDM and NDM alone were the three most common genotypes of carbapenemases observed during both the phases of the study. The percentage of isolates carrying NDM decreased from 56 to 43% from the Pre-COVID to COVID phase, but isolates with OXA-48 remained same at roughly 61% during both the study periods. Isolates harboring the other three carbapenemases KPC, VIM and IMP were few and accounted for less than 10 samples each. In *K. pneumoniae*, which was the most dominant species, OXA-48 alone or in combination with NDM were the most commonly reported carbapenemases. The same was true for *A. baumannii.* In the other two species, *E. coli* and *P. aeruginosa* NDM was the most common carbapenemase. Of the seven VIM positive isolates reported, 4 were from *P. aeruginosa*. The detailed distribution of the five carbapenemases in the four organisms is depicted in Fig. 2A and B.

The variants of NDM were determined by sequencing in 19 NDM positive isolates from both the study periods. These NDM PCR products sequenced were reported from 15 K*. pneumoniae*, 2 *E. coli* and 1 isolate each of *P. aeruginosa* and *A. baumannii* in each phase. NDM-5 was the most common variant during both the phases comprising 17 out of 20 samples in the first phase and 15 out 20 in the second phase. In the first phase all the 15 NDM isolated from *K. pneumoniae* had NDM-5, while in the second phase, 11 had NDM-5 and four reported NDM-1.The *A. baumannii* isolates from both the phases reported NDM-1, while in case of *P. aeruginosa* NDM-1 was detected in the first phase and NDM-5 in the second phase. The *E. coli* isolates reported NDM-5 in both the phases. In the first phase of the study, seven of the nine OXA-48 sequenced were OXA-232 and two OXA-181, while in 2021 there were five isolates carrying OXA-232, three had OXA-181 and one had OXA-48.

Conjugation assay was done in few selected isolates using azide resistant *E. coli* strain J53 as the recipient. Results of conjugation showed that the trans-conjugants were positive for NDM and OXA-48 indicating their presence on plasmids. The NDM, OXA-48 family sequences were submitted to Genbank, NCBI and have been assigned the following accession IDs (OM144483–OM144490), (OM164067–OM164096), (OM368081–OM368095) (OM458084–OM458086).

### Backbone of NDM containing plasmids

**Fig. 3:**
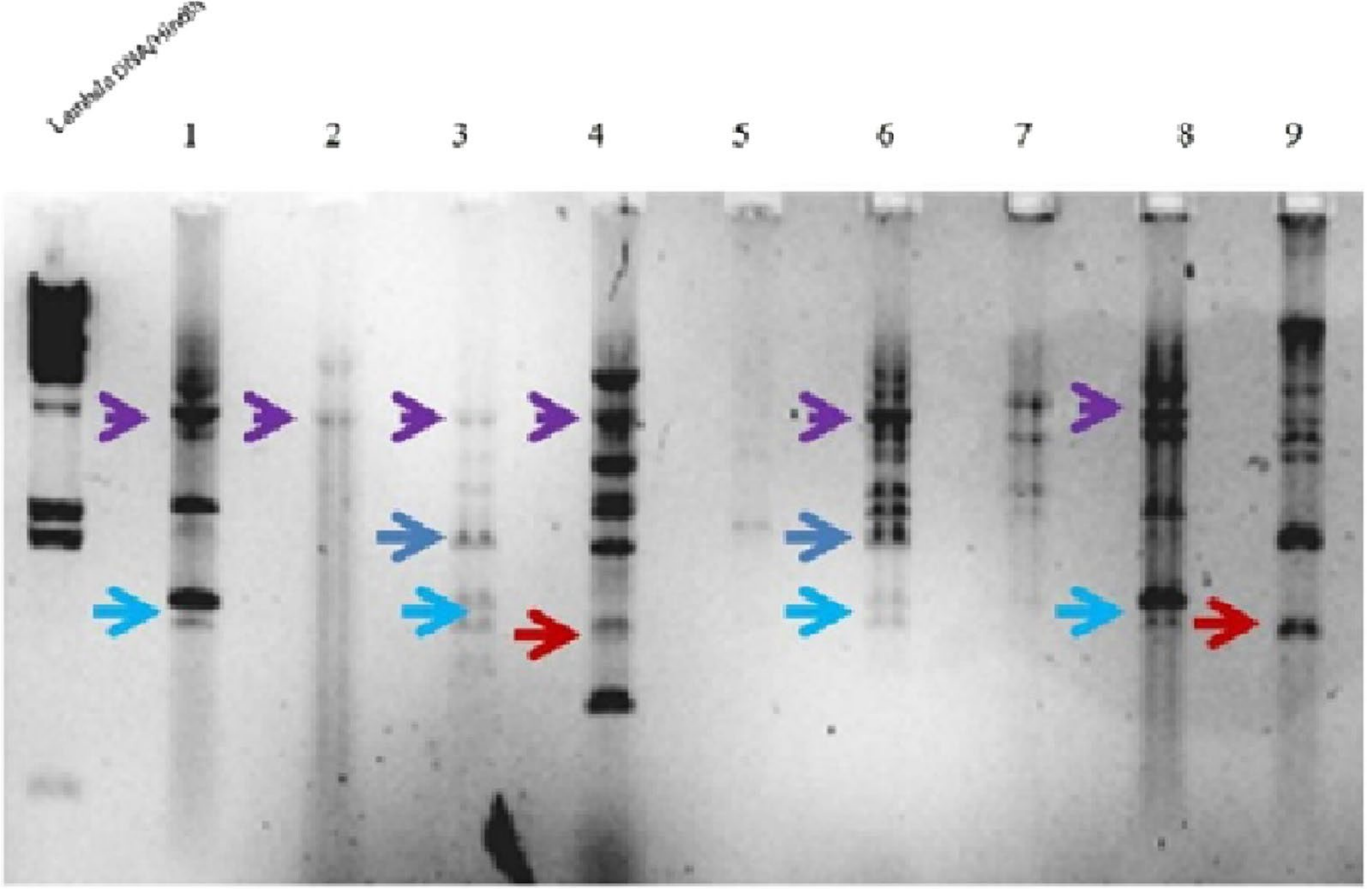
Plasmid profile of NDM carrying isolates by EcoR1 digestion. Gel picture of EcoR1 restriction digestion pattern of NDM carrying plasmids from the second phase (January to February 2021) of the study. Bands marked by arrows of the same colour indicate similar digestion pattern

In order to understand whether the plasmid background of the NDM genes was similar, we performed restriction digestion using EcoR1 of plasmids from 9 isolates carrying NDM. The restriction digestion gel picture is shown in Fig.3. Careful observation of the gel picture shows that samples in lanes 1 and 8 are highly similar. Likewise, samples in lane 3 and 6 also have a similar restriction digestion pattern, while the samples in lane 4 and 9 despite certain percentage of similarity are largely different from the rest of the plasmids. Although, the digestion pattern was not exactly similar, there was a certain similarity between the plasmids, indicating the transmission of plasmids within the hospital set-up.

## Discussion

Antibiotic resistance has become a major impediment in the successful treatment of infections, surgical procedures, organ transplant and many sensitive medical interventions. During the COVID-19 pandemic, secondary bacterial and fungal infections have markedly increased the mortality rate of patients infected with SARS-CoV-2. An Indian study shows that majority of these infections were due to gram negative bacteria, of which *Acinetobacter baumannii* and *Klebsiella pneumoniae* showed a very high percentage of carbapenem resistance [13].

Through the present work we analyze the effect of COVID-19 pandemic on the carbapenem resistance rate in our hospital setting that is a multi-specialty hospital based in North India. Firstly, we observed an increase in the carbapenem resistance rates in all the organisms except for *E. coli* during the COVID times. Interestingly, in the early days of the COVID pandemic in India, during May to June 2020, the carbapenem resistance rates had fallen to half of pre-COVID times in our hospital (Additional file 1: Figure S1). Drastic decrease in patient footfall due to stringent lockdown and stricter adherence to infection control protocols like hand hygiene are the most probable reason for the decrease in resistance in the initial COVID days. In the later phase of COVID, although adherence to infection control protocols as reflected by compliance to hand hygiene remained high, but there was significant rise in patients with co-morbidities, who are more susceptible to resistant species. The fact that there is increase in the number of respiratory samples during the COVID times, which includes broncho-alveolar lavage and tracheal aspirate also reflects that there were more critical patients who were either suffering from respiratory illness or required invasive devices like intubation. Co-morbid condition is an independent risk factor for acquisition of carbapenem resistant Enterobacteriaceae [14]. Similarly, long term care and hospital stay, use of invasive devices increase the chance of acquiring carbapenem resistant organism; however the exact interplay of co-morbid conditions and transmission of resistant bugs within a facility remains unclear [15]. Thus, we presume that admission of severely ill patients with co-morbidities could be a primary reason for the increased resistance rates. Our data also suggests that COVID and related complications also increase the susceptibility to carbapenem resistance probably due to immune-compromised conditions and longer stay in hospitals.

The prevalence of different carbapenemases broadly remained the same during the two time periods with NDM, OXA-48 family and the co-expression of both the genes being the most common. However, there was decrease in isolates having NDM, while organisms expressing OXA-48 family remained at the same level. There was also an increase in VIM expressing isolates in the second phase of the study. In 2019, just one organism with VIM was screened, while in 2021 six VIM producers were identified. Approximately, 10% and 12% of isolates in the two time periods respectively did not have any of the five carbapenemases. Majority of these isolates were of *P. aeruginosa*. It has been reported earlier that downregulation of porin gene OprD and over-expression of efflux pumps could result in carbapenem resistance in *P. aeruginosa* even in absence of carbapenemases [16, 17].

Sequencing of the PCR products of NDM showed that NDM-5 and OXA-181/232 were the common variants of NDM and OXA-48 family in our sample. We have reported the dominance of NDM-5 in our hospital in our previous work [9]. Similar results have emerged from other Indian studies [18, 19]. In addition, we note that NDM-5 was reported from varied organism *K. pneumoniae*, *E. coli and P. aeruginosa* indicating inter-species transmissibility. Four NDM-5 isolates were reported from patients of foreign origin coming from Ethiopia, Turkmenistan and Sudan. In absence of initial culture during admission, it is difficult to conclude whether they acquired the genes during their course of stay in the hospital.

OXA-232 was the most common variant of the OXA-48 family followed by OXA-181. Conjugation assay showed that both the NDM and OXA-48 family genes were plasmid based as trans-conjugants were positive for these genes. A recent report from India showed that plasmids carrying OXA-48 family genes OXA232/181 were present in small non-conjugative plasmids and were transmissible only in the presence of conjugative plasmids of NDM-5 [20]. To have a fair idea of the plasmid background, we next analyzed the plasmids of few isolates containing NDM by restriction digestion with enzyme EcoR1. The restriction digestion of plasmids showed more than one pattern indicating presence of different genetic background, replicon types or variation in the number of plasmid based genes. Similar observations have been made in NDM gene carrying plasmids originating from the same hospital or region [21, 22]. In absence of whole genome sequencing we cannot ascertain the genetic background of the NDM and OXA-48 genes. The non-uniform restriction digestion pattern between the isolates gives an indication that the NDM genes might be either present in different plasmid types that may have entered the hospital at different time points or have undergone microevolution in the hospital set-up. Such incidences of microevolution have been monitored using Single molecule real-time sequencing (SMRT) in OXA-48 producing *Klebsiella pneumoniae* in a previous study [23].

The study analyses the effect of pandemic COVID-19 on the rate of carbapenem resistance from northern India, which is considered an endemic region. Such studies are few and therefore the findings crucial for prevention of infection and policy making. Yet, the study suffers from certain limitations. One of the weaknesses was our inability to conduct the study in three consecutive years, which would have given us a clear idea of the trends in carbapenem resistance rates and prevalence of difference carbapenemases in the pre and post lockdown periods. Secondly, we have not been able to conduct the molecular testing on all carbapenem resistant isolates. Finally, to establish the clonal or genetic relatedness between the isolates and plasmids, whole genome sequencing is the best technique, which we could not conduct due to large sample size and cost issues.

## Conclusion

Taken together, we conclude that carbapenem resistance has shown an increasing trend in COVID times in the dominant species *K. pneumoniae*. The increase in resistance was primarily due to the rise of number of patients with co-morbidities. The genetic elements of carbapenem resistance have largely remained the same.

## Supplementary Information

**Additional file 1: Figure S1.** Carbapenem resistance rates in Pre-COVID and Initial COVID times. Carbapenem resistance rates in the four gram negative species under study: *E. coli*, *K. pneumoniae*, *A. baumannii* and *P. aeruginosa* during Early COVID (Jan-Feb 2020 and May-Jun 2020). The second phase coincided with stringent lockdown time [EC: *E. coli*, KP: *K. pneumoniae*, AB: *A. baumannii*, PA: *P. aeruginosa*].

## Data Availability

The datasets analysed during the current study are available from the corresponding author on reasonable request.

## Abbreviations

NDM: New Delhi metallo-β-lactamase
OXA-48: Oxacillinase-48
VIM: Verona integron-encoded metallo-β-lactamase
KPC: Klebsiella pneumoniae carbapenemase
IMP: *Imipenase*
ND: None of the tested carbapenemase detected

## References

[1] Chandy SJ, Thomas K, Mathai E, Antonisamy B, Holloway KA, Stalsby LC (2013). Patterns of antibiotic use in the community and challenges of antibiotic surveillance in a lower-middle-income country setting: a repeated cross-sectional study in Vellore, South India. J Antimicrob Chemother.

[2] Farooqui HH, Selvaraj S, Mehta A, Heymann DL (2018). Community level antibiotic utilization in India and its comparison vis-à-vis European countries: evidence from pharmaceutical sales data. PLoS ONE.

[3] Livermore DM (2021). Antibiotic resistance during and beyond COVID-19. JAC Antimicrob Resist..

[4] Ukuhor HO (2021). The interrelationships between antimicrobial resistance, COVID-19, past, and future pandemics. J Infect Public Health.

[5] Bush K, Bradford PA (2020). Epidemiology of β-lactamase-producing pathogens. Clin Microbiol Rev.

[6] Veeraraghavan B, Walia K (2019). Antimicrobial susceptibility profile & resistance mechanisms of Global Antimicrobial Resistance Surveillance System (GLASS) priority pathogens from India. Indian J Med Res.

[7] Collee JG, Miles RS, Wan B, Collee JG, Fraser AG, Marmion BP, Simmons A (1996). Tests for the identification of bacteria. Mackie and McCartney practical medical microbiology.

[8] Clinical and Laboratory standards Institute [CLSI]: Performance Standards for Antimicrobial Susceptibility Testing, 28th Informational Supplement M100. CLSI, Wayne, PA, 2006.

[9] Jaggi N, Chatterjee N, Singh V, Giri SK, Dwivedi P, Panwar R, Sharma AP (2019). Carbapenem resistance in *Escherichia coli* and *Klebsiella pneumoniae* among Indian and international patients in North India. Acta Microbiol Immunol Hung.

[10] Dallenne C, Da Costa A, Decré D, Favier C, Arlet G (2010). Development of a set of multiplex PCR assays for the detection of genes encoding important beta-lactamases in Enterobacteriaceae. J Antimicrob Chemother.

[11] Wang M, Tran JH, Jacoby GA, Zhang Y, Wang F, Hooper DC (2003). Plasmid-mediated quinolone resistance in clinical isolates of *Escherichia coli* from Shanghai, China. Antimicrob Agents Chemother.

[12] Benz F, Huisman JS, Bakkeren E (2021). Plasmid- and strain-specific factors drive variation in ESBL-plasmid spread in vitro and in vivo. ISME J.

[13] Vijay S, Bansal N, Rao BK, Veeraraghavan B, Rodrigues C, Wattal C, Goyal JP, Tadepalli K, Mathur P, Venkateswaran R, Venkatasubramanian R, Khadanga S, Bhattacharya S, Mukherjee S, Baveja S, Sistla S, Panda S, Walia K (2021). Secondary infections in hospitalized COVID-19 patients: Indian experience. Infect Drug Resist.

[14] Miller BM, Johnson SW (2016). Demographic and infection characteristics of patients with carbapenem-resistant Enterobacteriaceae in a community hospital: development of a bedside clinical score for risk assessment. Am J Infect Control.

[15] van Duin D, Perez F, Rudin SD, Cober E, Hanrahan J, Ziegler J, Webber R, Fox J, Mason P, Richter SS, Cline M, Hall GS, Kaye KS, Jacobs MR, Kalayjian RC, Salata RA, Segre JA, Conlan S, Evans S, Fowler VG, Bonomo RA (2014). Surveillance of carbapenem-resistant *Klebsiella pneumoniae*: tracking molecular epidemiology and outcomes through a regional network. Antimicrob Agents Chemother.

[16] Muderris T, Durmaz R, Ozdem B, Dal T, Unaldı O, Aydogan S, Celikbilek N, Acıkgoz ZC (2018). Role of efflux pump and OprD porin expression in carbapenem resistance of Pseudomonas aeruginosa clinical isolates. J Infect Dev Ctries.

[17] Khalili Y, Yekani M, Goli HR, Memar MY (2019). Characterization of carbapenem-resistant but cephalosporin-susceptible *Pseudomonas aeruginosa*. Acta Microbiol Immunol Hung.

[18] Paul D, Babenko D, Toleman MA (2020). Human carriage of cefotaxime-resistant *Escherichia coli* in North-East India: an analysis of STs and associated resistance mechanisms. J Antimicrob Chemother.

[19] Devanga Ragupathi NK, Veeraraghavan B, Muthuirulandi Sethuvel DP, Anandan S, Vasudevan K, Neeravi AR, Daniel JLK, Sathyendra S, Iyadurai R, Mutreja A (2020). First Indian report on genome-wide comparison of multidrug-resistant *Escherichia coli* from blood stream infections. PLoS ONE.

[20] Naha S, Sands K, Mukherjee S, Saha B, Dutta S, Basu S (2021). OXA-181-like Carbapenemases in *Klebsiella pneumoniae* ST14, ST15, ST23, ST48, and ST231 from septicemic neonates: coexistence with NDM-5, resistome, transmissibility, and genome diversity. mSphere.

[21] Ahmad N, Khalid S, Ali SM, Khan AU (2018). Occurrence of *bla*_NDM_ variants among enterobacteriaceae from a neonatal intensive care unit in a Northern India Hospital. Front Microbiol.

[22] Hirabayashi A, Yahara K, Mitsuhashi S, Nakagawa S, Imanishi T, Ha VTT, Nguyen AV, Nguyen ST, Shibayama K, Suzuki M (2021). Plasmid analysis of NDM metallo-β-lactamase-producing Enterobacterales isolated in Vietnam. PLoS ONE.

[23] Zautner AE, Bunk B, Pfeifer Y, Spröer C, Reichard U, Eiffert H, Scheithauer S, Groß U, Overmann J, Bohne W (2017). Monitoring microevolution of OXA-48-producing Klebsiella pneumoniae ST147 in a hospital setting by SMRT sequencing. J Antimicrob Chemother.

