# Supplementary figures and images for: Trends in carbapenem resistance in Pre-COVID and COVID times in a tertiary care hospital in North India
Source: Ann Clin Microbiol Antimicrob. 2023 Jan 3;22:1. doi: 10.1186/s12941-022-00549-9 (PMC9808733; doi:10.1186/s12941-022-00549-9)

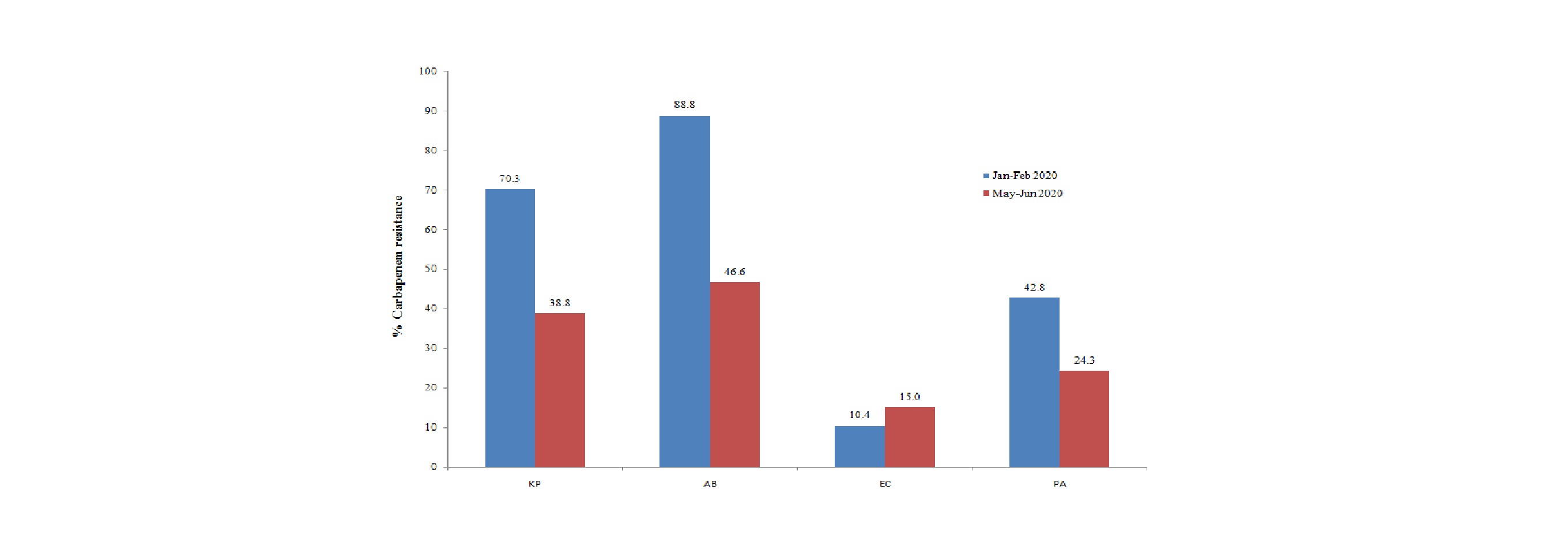

Supplement: Supplementary file 1 — Additional file 1: Figure S1. Carbapenem resistance rates in Pre-COVID and Initial COVID times. Carbapenem resistance rates in the four gram negative species under study: E. coli, K. pneumoniae, A. baumannii and P. aeruginosa during Early COVID (Jan-Feb 2020 and May-Jun 2020). The second phase coincided with stringent lockdown time [EC: E. coli, KP: K. pneumoniae, AB: A. baumannii, PA: P. aeruginosa]. [file 12941_2022_549_MOESM1_ESM.jpg]
